# A focus on substituents effect in the force-promoted disrotatory ring-opening of *cis*-cyclobutenes

**DOI:** 10.1039/d5sc90082d

**Published:** 2025-04-15

**Authors:** Lei Chen, Guillaume De Bo

**Affiliations:** a Department of Chemistry, University of Manchester Manchester M13 9PL UK Guillaume.debo@manchester.ac.uk

## Abstract

Symmetry-forbidden reactions are notoriously difficult to investigate as they are typically overshadowed by the corresponding symmetry-allowed pathway. Mechanical activation allows access to reaction pathways disfavoured using other methods of activation, such as the symmetry-forbidden disrotatory ring-opening of substituted *cis*-cyclobutenes. In a recent publication, Bowser, *et al.* have studied the effects of various substituents on this reaction using atomic force microscopy and computational analysis (B. H. Bowser, C. L. Brown, J. Meisner, T. B. Kouznetsova, T. J. Martínez and S. L. Craig, *Chem. Sci.*, 2025, https://doi.org/10.1039/D5SC00253B). The largest effect is observed with substituents close to the scissile bond having the ability to stabilise the diradical character of the disrotatory ring-opening reaction pathway.

## Abstract 2

Bowser *et al.* have shown that substituents stabilising the diradical character of the symmetry-forbidden disrotatory pathway accelerate the mechanochemical ring-opening of *cis*-cyclobutenes (*Chem Sci.*, 2025, https://doi.org/10.1039/D5SC00253B).

## Graphical abstract

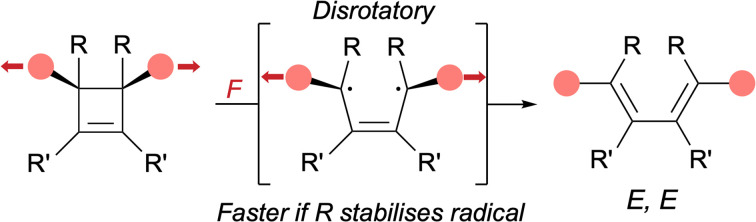

In polymer mechanochemistry, polymers are used to activate force-sensitive molecules (mechanophores),^1^ sometimes along unusual reaction pathways. The most striking example of such unique reactivity is perhaps the symmetry-forbidden disrotatory ring-opening reaction of *cis* cyclobutene (CBE) and benzocyclobutene (BCB) mechanophores (Fig. 1). The first example was reported by Moore and coworkers in a landmark paper demonstrating the anti-Woodward-Hoffman ring-opening of BCB.^2^ This seminal report was quickly followed by computational and single-molecule studies of BCB and CBE by the groups of Marx, Martínez, and Craig.^3–8^

**Fig. 1:**
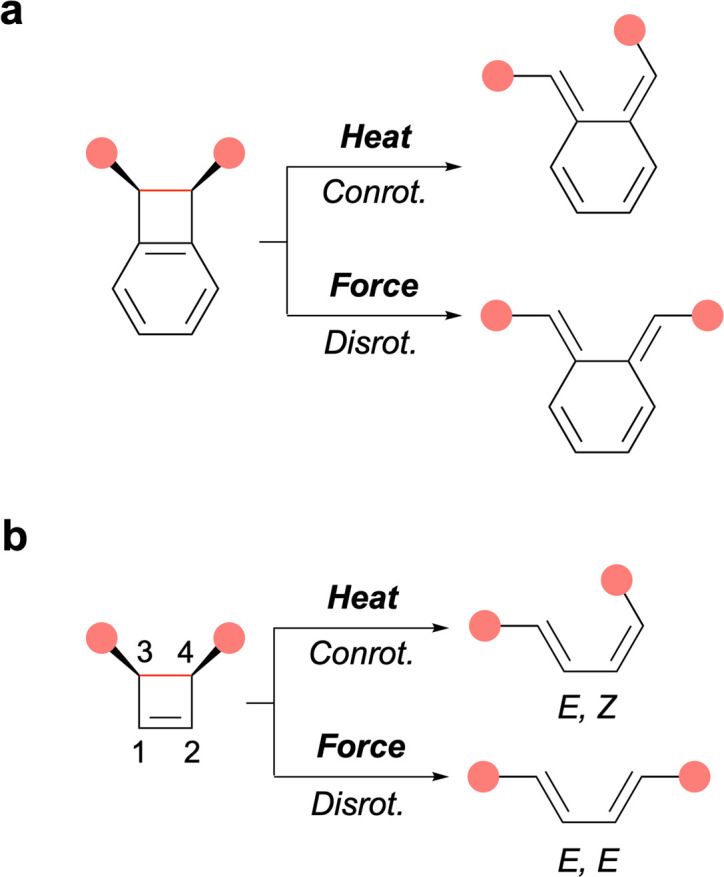
Thermal and mechanical activation of *cis*-BCB (a) and *cis*-CBE (b) mechanophores, leading to conrotatory and disrotatory ring-opening reactions, respectively. Pink disks indicate the position of the pulling points. Scissile bonds shown in red.

Mechanical activation offers a unique opportunity to study the disrotatory reaction pathway in more detail, for example to investigate substituent effects, as it is mostly inaccessible to thermal activation. Previously, Craig and Martínez have compared the forbidden/allowed ring-opening of *cis/trans* BCB and CBE mechanophores, differing by the presence of a fused (BCB) or connected (CBE) phenyl ring(s) at positions 1 and 2 of the 4-membered ring core (Fig. 1). They found that BCB and CBE respond in contrasting ways to mechanical force as the forbidden ring-opening of *cis*-BCB occurs at lower force than the allowed ring-opening of *trans*-BCB, while the opposite is true for *cis*- and *trans*-CBE.^8^

In a recent article published in this journal, the Craig and Martínez groups teamed up again (https://doi.org/10.1039/D5SC00253B) to investigate the effect of substituents in positions 1,2 and 3,4 of various CBE mechanophores using atomic force microscopy (AFM) in concert with computational methods (Fig. 2).^9^

**Fig. 2:**
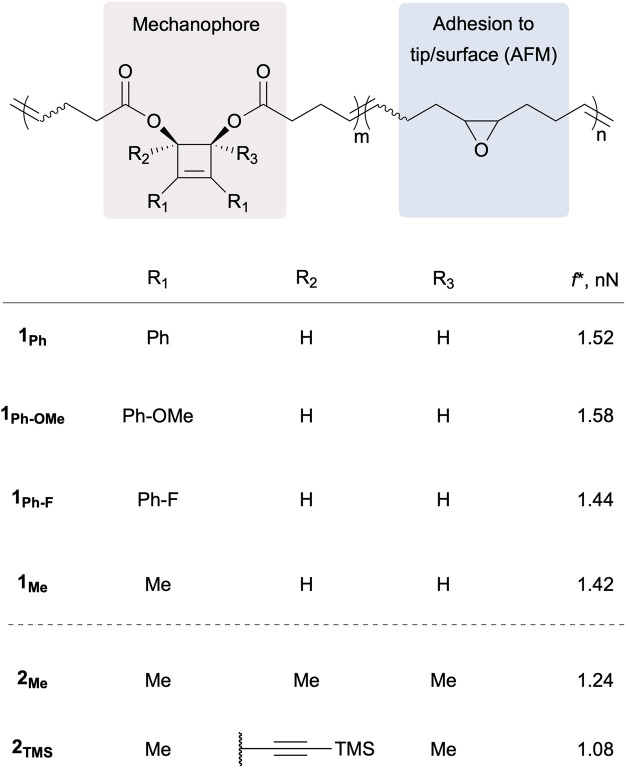
Copolymers containing CBE mechanophores with various substituents and epoxides for adhesion to the tip/surface of the AFM setup, and their activation force (f*) determined by AFM.

First, the effect of substitution at positions 1 and 2, which are remote from the scissile bond, was investigated on multimechanophore polymers 1_Ph–X_ possessing phenyl groups of varying electronic density (X = H, OMe, F) at these positions. All three polymers exhibit a similar activation force (f*) as measured by AFM (Fig. 2). The low level of influence from substituents at these positions was further confirmed with 1_Me_, which displays an activation force in the same range. In previously investigated systems, the activation of a CBE mechanophore is believed to proceed *via* both disrotatory and conrotatory pathways at low force, with the disrotatory reaction channel being predominant at higher forces.^8^ This picture was confirmed in the present study from the activation energy calculated for the range of forces recorded by AFM. This means that the ring-opening could theoretically proceed *via* a symmetry-allowed thermal conrotatory process, leading to an *E*,*Z* butadiene, or *via* the concurrent force-driven disrotatory pathway to afford the *E*,*E* product (Fig. 1). The subtle difference in contour length between the 2 butadiene isomers was matched with the elongation plateaus recorded in the AFM profiles to conclude that the disrotatory pathway was predominant in these experiments.

Substituents at positions 3 and 4, adjacent to the scissile bond, have a much greater effect as determined from polymers 2_Me_, possessing Me groups in all four positions, and 2_TMS_, in which one of the Me group is replaced by a trimethylsilylacetylene (TMSA) moiety. The same contour length analysis was used to confirm that these polymers also react predominantly *via* a disrotatory mechanism to produce the longer *E*,*E* isomer. However, these ring openings occur at much lower forces (f*) than in polymers 1_x_ (Fig. 2), indicating a lower activation barrier for this disrotatory process. A mechanophore’s reactivity can be assessed from its mechanochemical coupling (*i.e.* how efficiently force affects the activation energy) and its intrinsic (*i.e.* force-free) reactivity along a specific reaction pathway. Despite their varied f* values, all the CBE mechanophores show a similar mechanochemical coupling, which is inherent to the *cis* geometry of the core CBE structure. In contrast, the presence of Me, and even more of TMSA, increases the intrinsic reactivity of these CBE mechanophores compared to their unsubstituted counterpart (1_Me_). As the disrotatory pathway displays a significant diradical character,^8^ the difference in reactivity is mainly attributed to the ability of these groups to stabilise the developing radicals in positions 3 and 4. Computational analysis even allowed the weighing of the stabilising contribution of each group (1.5–2 and 4.5–6.5 kcal per mol per Me and TMSA groups respectively). Interestingly, 2_TMS_ proved to be mechanochromic as the TMSA group imparts the corresponding ring-opened product with fluorescence–emissive properties due to the conjugation of TMSA with the butadiene backbone, as demonstrated by sonication.

By combining single force spectroscopy and computational techniques, the authors were able to investigate the symmetry-forbidden disrotatory ring-opening of CBE mechanophores in great detail. Notably, they were able to identify the mechanical signature of the disrotatory process and isolate the effect of substituents on the ring-opening, which supports the diradical character of the disrotatory mechanism. The competition between concerted and radical mechanisms has been invoked in many force-promoted pericyclic reactions and the latter is often considered dominant in the nN regime.^10^ However, even though it’s been shown computationally,^10,11^ there are few experimental validations of the radical mechanism.^12^ The possibility of probing these competing mechanisms, and underlying effects, by AFM is an exciting prospect and would help shed light on the reactivity of mechanophores at low force and strain rate, and near the mechanistic cross over point. This study further highlights the unique position occupied by the field of polymer mechanochemistry in the study of reaction pathways inaccessible to other methods of activation.

## Author contributions

L. C. and G. D. B. co-wrote the manuscript.

## Conflicts of interest

There are no conflicts to declare.

## References

[cit1] De Bo G. (2020). Macromolecules.

[cit2] Hickenboth C. R., Moore J. S., White S. R., Sottos N. R., Baudry J., Wilson S. R. (2007). Nature.

[cit3] Ribas-Arino J., Shiga M., Marx D. (2009). Chem.–Eur. J..

[cit4] Ong M. T., Leiding J., Tao H., Virshup A. M., Martínez T. J. (2009). J. Am. Chem. Soc..

[cit5] Wang J., Kouznetsova T. B., Niu Z., Ong M. T., Klukovich H. M., Rheingold A. L., Martínez T. J., Craig S. L. (2015). Nat. Chem..

[cit6] Wang J., Kouznetsova T. B., Niu Z., Rheingold A. L., Craig S. L. (2015). J. Org. Chem..

[cit7] Bowser B. H., Wang S., Kouznetsova T. B., Beech H. K., Olsen B. D., Rubinstein M., Craig S. L. (2021). J. Am. Chem. Soc..

[cit8] Brown C. L., Bowser B. H., Meisner J., Kouznetsova T. B., Seritan S., Martínez T. J., Craig S. L. (2021). J. Am. Chem. Soc..

[cit9] Bowser B. H., Brown C. L., Meisner J., Kouznetsova T. B., Martínez T. J., Craig S. L. (2025). Chem. Sci..

[cit10] Akbulatov S., Boulatov R. (2017). ChemPhysChem.

[cit11] Cardosa-Gutierrez M., De Bo G., Duwez A.-S., Remacle F. (2023). Chem. Sci..

[cit12] Kean Z. S., Niu Z., Hewage G. B., Rheingold A. L., Craig S. L. (2013). J. Am. Chem. Soc..

